# Of Bloodletting in Pneumonia

**Published:** 1853-06

**Authors:** 


*Of Blood-letting in Pneumonia.*—The memoir of Dietl on this subject, published several years ago, excited much attention at the time, and we think it will not be unprofitable to transcribe some of the facts and conclusions contained in a recent report by the same author, inserted in the weekly medical journal of Vienna (*Wein. Med. Voschr.*) This memoir comprises all the cases of primitive and essential pneumonia treated at the great hospital of Vienna from 1847 to 1850, without excepting those aggravated by age, regimen, or analogous circumstances. Cases of secondary pneumonia supervening upon acute or chronic diseases, such as typhus, Bright's disease, etc., are excluded. No patient has been bled in pneumonia in this hospital since 1844; the practice is limited to draughts of gum-water, or ptisans containing opium or an expectorant.

In three years, 750 cases of pneumonia were treated on the expectant plan, viz.: 412 men, 338 women. Of these the pneumonia was complicated with acute disease in 140 cases, and with chronic disease in 249. Hyperæmia of the liver and acute intestinal catarrh were the most frequent complications; meningitis was never observed; gangrene of the lung only twice, and pulmonary abscess in a single case. Of 750 patients treated without blood-letting, 681 recovered, viz.: 384 men, 297 women; 69 died: 28 men, 41 women—8 died in the stage of red hepatization; 56 in that of infiltration; 5 in that of purulent infiltration. No fatal case was exempt from complication. The mean duration of the disease was 20 or 21 days. In 515 cases the dyspnœa was very great; it is incontestible that the abstraction of blood diminishes the oppression, but the relief is only temporary. The author admits, however, that when the pneumonia is abandoned to the expectant plan, the embarrassment of the respiration is almost intolerable. This is, he adds, an inconvenience for which the rapid convalescence more than compensates.—*Archives Generales*, (Jan. 1852.)—*From Virginia Med. and Surg. Journ.*

